# Virtual and in-person cardiac rehabilitation

**DOI:** 10.1136/bmj.n1270

**Published:** 2021-06-03

**Authors:** Hasnain M Dalal, Patrick Doherty, Sinead TJ McDonagh, Kevin Paul, Rod S Taylor

**Affiliations:** 1 University of Exeter Medical School, Royal Cornwall Hospital, Truro, UK; 2 Primary care Research Group, University of Exeter Medical School, St Luke’s Campus, Exeter, UK; 3 Department of Health Sciences, University of York, York, UK; 4 REACH-HF Patient and Public Involvement Group, c/o Research & Development, Royal Cornwall Hospitals NHS Trust, Truro, UK; 5 MRC/CSO Social and Public Health Sciences Unit & Robertson Centre for Biostatistics, Institute of Health and Wellbeing, University of Glasgow, Glasgow, UK

**What you need to know**

- Most eligible patients with coronary heart disease and heart failure do not participate in cardiac rehabilitation. Covid-19 has exacerbated this, with a substantial drop in the number of patients participating
- Home and telehealth based interventions are increasingly being used as alternatives to traditional centre based rehabilitation programmes
- Outcomes for patients participating in home based rehabilitation compare favourably with centre based programmes in terms of hospitalisations, quality of life, and cost
- Telehealth based interventions are promising, but some patients may find these interventions challenging
- Novel ways of delivering rehabilitation have been employed during the covid-19 pandemic, including hybrid models that are likely to be offered as alternatives to centre based rehabilitation in future, enabling greater patient choice and greater uptake of cardiac rehabilitation

Before the covid-19 pandemic, 100 000 people were admitted to hospital with heart attacks and approximately 200 000 were diagnosed with heart failure annually in the UK.^1^ An estimated 7.4 million people in the UK live with cardiovascular diseases, and this is likely to increase with improved survival following coronary heart disease and an ageing population.^1^

A 2020 European position paper, in keeping with other national and international guidelines,^2^ ^3^ stated that “comprehensive cardiac rehabilitation has been recognised as the most cost effective intervention to ensure favourable outcomes across a wide spectrum of cardiovascular disease.”^4^ Benefits include improvements in morbidity, hospital admissions, physical activity, exercise capacity, psychological wellbeing, and health related quality of life.^3^ ^4^ ^5^ ^6^ ^7^ ^8^ ^9^ ^10^ ^11^ Patient groups set to benefit are categorised, by evidence level, in box 1. To achieve these benefits, it is recommended that all core components of cardiac rehabilitation (box 2) are included in a comprehensive programme.^4^ Guidelines from the UK also advocate long term strategies to promote secondary prevention in primary care and service evaluation through audit.^3^

Box 1 **Patient groups who benefit from cardiac rehabilitation^4^**

**High level evidence**

Post-acute coronary syndrome (ACS), post-primary coronary angioplasty, and coronary artery surgery:
  Patients with ACS (class 1, level A,^12^ ^13^ including ST-segment elevation myocardial infarction, non-ST-segment elevation myocardial infarction, and unstable angina (class 1, level B^12^)
  All patients undergoing reperfusion (eg, coronary artery bypass graft, primary percutaneous coronary intervention, and percutaneous coronary intervention (class 1, level A^12^)
Chronic heart failure:
  Patients with newly diagnosed chronic heart failure and chronic heart failure with a step change in clinical presentation (class 1, level A^10^ ^11^)

**Limited evidence**

Patients with heart transplant and ventricular assist devices
Post-valve heart surgery (open and percutaneous)
Adults and adolescents with congenital heart disease
Atrial fibrillation

Unlike other international guidelines, evidence informing guidance from the National Institute for Health and Care Excellence is assessed based on Grading of Recommendations, Assessment, Development, and Evaluations (GRADE) criteria—the class/level approach is not used and therefore not referenced above

Box 2 **Core components of cardiac rehabilitation, according to broad international consensus^4^**

Patient assessment
  Clinical history
  Symptoms
  Physical examination
  Investigations: electrocardiogram, cardiac imaging, blood tests
  Physical activity level
  Peak exercise capacity—eg, bicycle ergometer, treadmill, validated walk test
Physical activity counselling
  Type and level
  Education
  Barriers to exercise
  Exercise for patients unable to engage in walking or cycling based activities
Exercise training
  Individualised prescribing
  Frequency, intensity, time (duration), and type of exercise (FITT), or FITT related to mealtime (FITT +T)
Diet/nutritional counselling
  Assessment
  Education
  Healthy food choices
Weight control management
  Assessment
  Education
  Weight reduction
Lipid management
  Assessment
  Diet, physical activity, and medication
Blood pressure management
  Assessment
  Lifestyle intervention and medication
Smoking cessation
  Smoking status, including other tobacco products
  Encouragement to stop smoking all tobacco
  Referral for smoking cessation
Psychosocial management
  Assessment of psychosocial risk factors
  Referral for behavioural and psychosocial intervention
  Vocational reintegration/return to work
Evaluation of programme results
  Determination of success and failure of interventions
  New rehabilitative goals
  Communication regarding continuing care
  Quality assurance using systematic registration
  Structured follow-up

Even before the covid-19 pandemic, most patients in high and low to middle income countries were not offered cardiac rehabilitation^5^ ^14^ and uptake was low. Lockdown measures owing to covid-19 have exacerbated this problem.^15^ ^16^ Provision and use of cardiovascular healthcare have decreased worldwide, with substantial numbers of patients in Europe and North America unable to access routine hospital care.^15^ ^16^ In the UK, covid-19 shielding guidance for patients with cardiovascular disease and redeployment of NHS staff to acute services have notably reduced access to and use of these services.^17^

This clinical update describes pre-pandemic evidence for cardiac rehabilitation and considers how adoption of a broader range of evidence based delivery methods can improve uptake and patient outcomes during the pandemic and beyond.

## Cardiac rehabilitation before the pandemic

Historically, rates of referral to cardiac rehabilitation were suboptimal in the US^18^ ^19^ and UK (<15% for heart failure).^20^ Uptake was also poor, with only 68 074 (50%) of the 135 861 patients with coronary heart disease in England, Wales, and Northern Ireland accepting an offer of cardiac rehabilitation in the 12 months before the pandemic.^21^

Most patients referred for rehabilitation after a cardiac event were offered supervised, group based classes, which ranged in frequency, intensity, duration of exercise, and self-help guidance.^4^ ^21^ Cardiac rehabilitation was usually delivered in hospital outpatient departments or community centres, or (in some parts of Europe) as inpatient services.^4^ Collectively, these modes of delivery are termed “centre based cardiac rehabilitation.” Despite compelling evidence for clinical and cost effectiveness, participation in centre based programmes remained suboptimal, with overall participation rates <20% in the US^22^ and similar rates after a diagnosis of heart failure in Europe.^23^ Poor participation predominated in certain groups: women, older people, ethnic minorities, and those living in rural communities or who are socioeconomically deprived.^4^ ^21^ ^24^

Consequently, calls were made for alternatives to centre based cardiac rehabilitation.^25^ Suggested interventions included rehabilitation at home facilitated by healthcare professionals and supported by telehealth technologies, to improve uptake.^26^ An American scientific statement in 2019 advocated home based cardiac rehabilitation,^9^ and guidance from the National Institute for Health and Care Excellence (NICE) on chronic heart failure in the UK in 2018 stated that “delivery of home based rehabilitation may increase access and uptake.^27^ Hybrid models involving a combination of home and centre based rehabilitation have been evaluated^28^ but not implemented widely.^9^ Tele-rehabilitation—“rehabilitation from a distance by using one or several devices monitoring and communicating patient specific information to the caregivers,”^29^ which often involves telephones, videoconferencing, and mobile apps (telehealth)^30^—is increasingly used, often as an adjunct to home based rehabilitation. 

Box 3 summarises key national and international recommendations on cardiac rehabilitation. Box 4 discusses ways to improve delivery of cardiac rehabilitation for patients, including under-represented populations.

Box 3 **Key recent recommendations on cardiac rehabilitation**

**World Health Organization**

In 2017, WHO issued a statement committing to key actions to strengthen cardiac rehabilitation services and highlighted the evidence indicating the benefits of rehabilitation after acute myocardial infarction.^31^ Rehabilitation should be “part of universal health coverage and should be incorporated into the package of essential services, along with prevention, promotion, treatment, and palliation”^31^

**UK**

The 2018 NICE guidance on chronic heart failure states that rehabilitation “should be provided in a format and setting (at home, in the community, or in the hospital) that is easily accessible for the person”^27^
The NHS Long Term Plan similarly underlines the importance of rehabilitation and has set an ambitious target of 85% of eligible patients being able to access care by 2028^32^
The importance of cardiac rehabilitation and delivering cardiac rehabilitation during the covid-19 pandemic is discussed in a cardiovascular disease impact report by NICE in 2021. https://www.nice.org.uk/Media/Default/About/what-we-do/Into-practice/measuring-uptake/cvd-impact-report/nice-impact-cvd-management.pdf

**US**

In response to the covid-19 outbreak, the American Association of Cardiovascular and Pulmonary Rehabilitation has established the Innovative Delivery Model Collaborative to facilitate digital approaches and encourage home based delivery of rehabilitation. It offers webinars on virtual cardiac and pulmonary rehabilitation. https://www.aacvpr.org/Learn/Learning-Center/Virtual-Rehab-Module-Series
A US scientific statement from 2019 noted that only a minority of eligible patients participate in rehabilitation and recommended home based interventions for low to moderate risk patients unable to access centre based programmes^9^
In 2016, the Million Hearts Cardiac Rehabilitation Collaborative of 100 organisations developed a road map to increase participation in rehabilitation from 20% to 70% by 2022^19^ ^24^

**Europe**

A 2020 European position paper focuses on centre based programmes but does not comprehensively consider home based interventions^4^ ^33^
A paper from the European Association of Preventive Cardiology on recent cardiac tele-rehabilitation studies provides a practical guide for the setup of rehabilitation services during the covid-19 pandemic^16^

**Covid-19**

The emergence of covid-19 has prompted calls for accelerated introduction of alternative methods of delivery that include home based and tele-rehabilitation options^15^ ^16^ ^30^ ^34^

Box 4 **Improving delivery of care for patients, including under-represented populations**

**Increasing referral to cardiac rehabilitation**

Referral to cardiac rehabilitation can be increased through automatic referral at discharge, which can be enhanced by informing the patient of the benefits of the intervention^18^ ^25^ ^35^ and by making referral a “quality of care indicator”^36^

**Addressing health inequalities**

Engagement with cardiac rehabilitation is determined by service level and patient level factors—for instance, age, sex, ethnicity, level of deprivation, timing of rehabilitation, and mechanism of referral^37^
  Economic evaluations of data from the British Heart Foundation National Audit of Cardiac Rehabilitation confirm uptake rates of 37.6% and 51.7% for the most and least deprived populations, respectively^37^
  Patients who are given a firm date to attend their initial rehabilitation assessment are more than four times more likely to engage in rehabilitation programmes^38^
Covid-19 has exacerbated health inequalities, with poor outcomes in marginalised populations, and has prompted calls to establish equitable care models “that build culturally appropriate communication and outreach practices to communicate with patients beyond traditional phone calls and office visits”^39^
Lower rates of digital literacy and access have also been reported in other marginalised groups during the pandemic.^39^ Differences in the digital literacy of the healthcare workforce also need to be considered.^40^ Further research on implementation of telehealth and home based approaches in cardiac rehabilitation is urgently needed to inform providers and commissioners.^29^ ^41^

## Cardiac rehabilitation during the pandemic

The number of patients with heart failure in the UK participating in rehabilitation decreased from 4969 (<10% of eligible patients) before the pandemic (May 2019-January 2020) to 1474 (<5% of eligible patients) during the first wave (February-August 2020).^42^ Analysis by the British Heart Foundation (BHF) published in 2020 mirrored other cardiac audits, showing a 30-40% decrease in use of cardiology and rehabilitation services because of the pandemic compared with a similar period in 2019.^17^ ^43^ Covid-19 has therefore led to further calls for alternatives to traditional centre based cardiac rehabilitation programmes, with an emphasis on home based and digital technologies to provide virtual access.^15^ ^16^

Indeed, although overall uptake of cardiac rehabilitation has decreased, the proportion of patients receiving home based rehabilitation in the UK has increased more than threefold since the pandemic—from 22.2% to 72.4%^42^—as more services began to offer home based and remote delivery.^17^ The rapid adoption of technology in response to suspension of centre based rehabilitation in the pandemic was also reported in an international survey of 330 cardiac rehabilitation healthcare professionals.^44^ Use of tele-rehabilitation can provide “a safe solution for patients, family, and staff in the midst of covid-19.”^30^ Examples of how programmes have adapted the delivery of rehabilitation in the pandemic have been shared by NICE.^45^ ^46^ Reporting by the BHF on modes of delivery in response to covid-19 now includes greater detail on 11 different cardiac rehabilitation interventions, including nine types of home based, virtual, and hybrid programmes.^17^

## How can exercise capacity be assessed remotely during the pandemic?

Baseline assessment of exercise capacity is core to the effective and safe delivery of exercise interventions.^3^ In the UK, the incremental shuttle walk test, step test, and six minute walk test are part of routine practice^3^ and are monitored by local programmes and reported nationally through the BHF audit. In response to covid-19, most cardiac rehabilitation programmes have adjusted assessments to include greater use of submaximal step tests, two and three minute walk tests, and other more subjective approaches, including physical activity questionnaires (eg, the Duke Activity Status index), fitness apps, and observation of patients carrying out activities such as chair based exercise or using hallways and stairs at home.

Several publications from North America and Europe discuss how to adapt and use different baseline fitness tests to assess the relative risk of exercise.^15^ ^16^ ^30^ ^47^ Risk assessment to determine whether patients have a fitness level below five metabolic equivalents and a proportional response (eg, heart rate and rating of exertion) to exercise is a key focus^15^ ^47^ ^48^ (box 5). Such changes are envisaged as temporary adjustments,^47^ as programmes had already started to reintroduce fitness tests before the January 2021 lockdown in the UK. However, robust studies of innovations—driven by covid-19—in home based assessment of exercise capacity could validate them for the future portfolio of exercise tests.

Box 5 **Practical tips for remote/virtual delivery of cardiac rehabilitation^15^**

Make it easy
  Use tip sheets to help staff adjust to delivering care virtually
  Avoid becoming overwhelmed by the multitude of available resources by finding a single, comprehensive, verified online resource for patients and staff
Don’t wait
  Encourage patients to attend at minimum intake assessments to discuss the merits of virtual cardiac rehabilitation
  Follow a shared decision making process for enrolment in virtual rehabilitation, to ensure patients understand potential risks and benefits of participating virtually versus choosing to delay care
Focus on core components*
  Consider lifestyle risk management, psychosocial support, medical advice, education
  Provide simple exercise prescriptions aimed at encouraging low-to-moderate physical activity
Obtain patient metrics
  Examples include a self-administered six minute walk test for exercise capacity, using patients’ personal scales and blood pressure cuffs
Offer group sessions
  Reduce “labour intensive” one-to-one sessions when possible by providing group tele-/video-conferencing for educational sessions and patient support
Evaluate
  Formalise an evaluation process to assess the merits and efficacy of virtual care
Invest in access
  For rural and/or under-resourced areas, consider purchasing tablets, smartphones, or other electronic options for loan to participants to enhance a one-to-one personal experience

*Moulson et al suggest that it is possible to complete assessment of the core components digitally/virtually, including an exercise test, albeit with the limitations^15^

## How do home and centre based cardiac rehabilitation programmes compare?

The UK, Australia, and Canada have been key adopters of home based cardiac rehabilitation.^9^ In the UK, the Heart Manual—acknowledged as the “most extensively studied self-management programme”^9^—has been used by the NHS for more than 10 years. Box 6 lists advantages and disadvantages of home based cardiac rehabilitation.

Box 6 **Advantages and disadvantages of home based versus centre based cardiac rehabilitation^9^**

**Potential advantages**

Reduced enrolment delays
Expanded capacity/access
Individually tailored programmes
Flexible, convenient scheduling
Minimal travel/transportation barriers
Greater privacy while receiving cardiac rehabilitation
Integration with regular home routine

**Potential disadvantages**

Lack of reimbursement*
Less intensive exercise training
Less social support
Less patient accountability
Lack of published standards for home based cardiac rehabilitation
Less face-to-face monitoring
Safety concerns for high risk patients

*Lack of reimbursement is an issue in some countries; however, the Centers for Medicare and Medicaid Services in the US introduced reimbursements for virtual cardiac rehabilitation in 2020^49^

The standards and core components (box 2) used in many home based cardiac rehabilitation studies are comparable with those in studies of centre based interventions).^9^ Cochrane reviews in coronary heart disease and heart failure consistently report statistically significant reductions in hospital admissions with centre and home based cardiac rehabilitation compared with usual care.^5^ In an updated Cochrane review that included 44 randomised controlled trials (10 home based and 34 centre based), the relative risk reduction for all cause hospitalisation was 0.70 (95% confidence interval (CI) 0.60 to 0.83)). Studies that directly or indirectly compared home and centre based cardiac rehabilitation found no statistically significant difference in 12 month mortality between the two approaches.^6^ ^7^ ^8^ ^9^ A systematic review of 31 randomised controlled trials reported that home based and hybrid cardiac rehabilitation models can improve exercise capacity and are potential alternatives to centre based programmes.^50^

A meta-analysis of individual participant data and the updated Cochrane review showed that participation in home or centre based cardiac rehabilitation after heart failure resulted in a clinically meaningful improvement in health related quality of life, determined using the Minnesota Living with Heart Failure questionnaire score (7.1 (95% CI -3.7 to -10.5)), compared with no rehabilitation.^6^ ^51^ Improvements in health related quality of life were reported for 11 of 13 trials of home versus centre based cardiac rehabilitation using various validated questionnaires, with no statistically significant difference between the two groups.^9^

**Fig 1:**
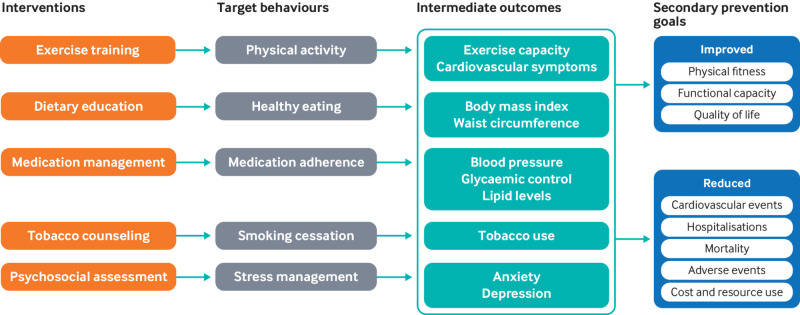
Structure, process, and outcome metrics for home based cardiac rehabilitation. Reproduced with permission^9^

Improvements in modifiable cardiovascular risk factors with cardiac rehabilitation are documented in a US scientific statement.^9^ Differential effects on weight, blood pressure, lipids, and tobacco use were similar in the eight home versus centre based cardiac rehabilitation trials that were included (fig 1).^9^

Adherence to home based cardiac rehabilitation is comparable with that in centre based programmes; however, considerable variation is seen in reporting of adherence, and the US scientific statement noted limited ability to pool data to compare home and centre based programmes.^9^ No statistically significant difference in adherence was reported between the two settings in a Cochrane review that included seven studies; however, three of the studies showed better adherence to home than centre based cardiac rehabilitation (RR 1.04 (95% CI 1.01 to 1.05); P=0.009).^7^ A small qualitative study nested within a randomised controlled trial reported that most patients, given the choice, prefer home based cardiac rehabilitation (57% versus 43%).^52^ Indeed, European and NICE guidelines recommend home based rehabilitation with or without telemonitoring, as evidence suggests that home based programmes can increase participation and support behaviour change.^2^ ^27^ A large prospective cohort study of 151 centres with 99 097 patients in the US reported that patients referred to home based cardiac rehabilitation were four times more likely to participate than those offered centre based programmes.^9^ ^53^ In response to covid-19, the Centers for Medicare and Medicaid Services in the US introduced reimbursements for virtual and home based cardiac rehabilitation from October 2020.^49^

A Cochrane review concluded that clinical benefits with centre and home based cardiac rehabilitation in patients with heart failure, myocardial infarction, and coronary revascularisation are similar and with equivalent costs.^7^ In this and the updated Cochrane review of rehabilitation for adults with heart failure, gains in health related quality of life and costs between home and centre based rehabilitation were also comparable.^6^ ^7^ Box 7 summarises the evidence for the cost effectiveness of home based programmes.

Box 7 **Cost effectiveness of home based cardiac rehabilitation and tele-rehabilitation**

A systematic review that examined economic evaluations in cardiac rehabilitation published since 2001 concluded that cardiac rehabilitation was cost effective compared with usual care^54^
  Incremental cost effectiveness ratios (ICERs) ranged from $1065 to $71 755 (£751 to £50 648; €869 to €58 557) per quality adjusted life year (QALY), although no significant differences in costs and QALYs were reported in two studies that directly compared home and centre based rehabilitation^54^
  Tele-rehabilitation was used in four included studies and was considered to be cost effective in all, but with wide ranging ICERs (from dominant (cardiac rehabilitation less costly and more effective than usual care) to $588 734 per QALY)^54^
  The authors called for larger studies to strengthen the evidence base, observing a potential reduction in costs if tele-rehabilitation were more widely adopted and noting that patient adherence to digital technologies is variable^54^
Health economic modelling based on a recent multicentre trial in patients with heart failure concluded that home based cardiac rehabilitation can be cost effective in the health service setting in the UK^55^
A facilitated home based cardiac rehabilitation intervention for people with heart failure and their caregivers: a research programme including the REACH-HF RCT^56^

## How do tele-rehabilitation and centre based cardiac rehabilitation programmes compare?

Tele-rehabilitation interventions are promising and are replacing centre based programmes as part of home based approaches during the pandemic.^30^ In a trial of 162 adults with coronary heart disease, which compared the effects of remotely monitored rehabilitation (by smartphone and web apps) with centre based programmes, remote cardiac rehabilitation was “an effective, cost efficient alternative delivery model that could improve overall utilisation rates by increasing reach and satisfying unique participant preferences.”^57^ However, caution is urged when implementing telehealth based interventions, as the mean age in most studies is less than 60 years. Some patients may find such interventions challenging without facilitated support.^4^ ^29^ ^41^ Ensuring quality assurance with telehealth based modes of delivery is also potentially challenging.^9^ ^16^ ^29^

A systematic review and meta-analysis that included 30 telehealth trials of secondary prevention in patients with coronary heart disease reported statistically significant lower re-hospitalisation and cardiac events (RR 0.56 (95% CI 0.39 to 0.81), P<0.0001) in the intervention groups. The authors concluded that telehealth interventions could be offered to patients unable to attend centre based rehabilitation.^58^

Another meta-analysis, which reviewed 11 trials (n=1189), reported that telehealth interventions were at least as effective as centre based programmes for improving modifiable cardiovascular risk factors and exercise capacity.^59^ Adherence to exercise was significantly higher with telehealth (fixed effect standardised mean difference 0.75 (95% CI 0.52 to 0.98)).^59^ Telehealth rehabilitation programmes can also quantify adherence to home based programmes through wearable accelerometer devices linked to the internet.^9^

## Conclusion

Poor rates of participation in conventional cardiac rehabilitation programmes led to the development of home based and digitally delivered interventions, which are supported by emerging evidence. Covid-19 has provided an opportunity “to reimagine how cardiac rehabilitation is delivered.”^49^ In the future, patients with cardiovascular disease are likely to be offered alternatives to centre based cardiac rehabilitation, including hybrid models, which will provide patient choice^52^ and should increase overall uptake in keeping with ambitious national targets in the US and UK.^19^ ^32^

**Education into practice**

- How well do you know the local pathways to refer patients for home based cardiac rehabilitation after a heart attack or new diagnosis of heart failure?
- In patients with a new diagnosis of heart failure, how many have been referred to and participated in a cardiac rehabilitation programme in the past 12 months?

**Ongoing research and unanswered questions**

**Ongoing research**

The National Institutes for Health Research (NIHR) Health Technology Assessment (HTA) Programme is funding a study based in the UK: A randomised controlled trial of a facilitated home based rehabilitation intervention in patients with heart failure with preserved ejection fraction and their caregivers: the REACH-HFpEF Study (2021-2024)^60^
The Western Norway Health Authority has funded a study of 3000 patients offered cardiac rehabilitation after a percutaneous coronary intervention,^61^ which will allow comparison of outcomes with another study on hospitalisation of older adults after acute myocardial infarction^62^: Rethinking rehabilitation after percutaneous coronary intervention: a multicentre cohort study on continuity of care, health literacy, adherence, and costs at all care levels (the CONCARDPCI) study^61^
The Improving ATTENDance in Cardiac Rehabilitation Trial (iATTEND) (2019-23)^63^: A randomised trial of 270 patients in the US to assess the efficacy of a hybrid approach to delivery of cardiac rehabilitation on attendance by combining both centre based and remote or home based cardiac rehabilitation sessions. The intervention group combines centre based and remote/home based cardiac rehabilitation and is tailored to the individual needs of each patient, accomplished with the assistance of an easy-to-access telecommunications method (telemedicine)

**Unanswered questions^4^ ^9^ ^64^**

What is the impact of
  home based services in more diverse and higher risk groups of patients?
  hybrid models of cardiac rehabilitation, including components from both centre and home based settings?
What are the barriers for using digital health technologies including wearable monitoring devices for cardiac rehabilitation in terms of factors that are
  patient related?
  clinician related?
  legal and ethical issues?
  interoperability and technical issues?
  lack of reimbursement?
What study designs should be used to evaluate multimorbidity cardiac rehabilitation programmes?
What is the feasibility of delivering cardiac rehabilitation through primary care networks?
Can we improve inequality in the uptake of cardiac rehabilitation by culturally adapting programmes for ethnic minority groups?

**How patients were involved in the creation of this article**

Cindy Edgeler is married to Chris, a retired roadman with Cornwall Highways, who was admitted to hospital with a heart attack in 2012 (Box “A carer’s story by Cindy Edgeler”). Chris was a patient of coauthor HD, and both Cindy and Chris were members of the Patient and Public Involvement Group that was part of the Rehabilitation Enablement in CHronic Heart Failure (REACH-HF) National Institute for Health Research (NIHR; RP-DG-0709-10111) funded clinical trial. Cindy and Chris reviewed a draft version of the clinical review.

We also received feedback from patients supported by the Wirral Community Cardiology Service, who have been delivering REACH-HF since 2019 and throughout the COVID-19 outbreak. https://www.nice.org.uk/sharedlearning/delivering-rehabilitation-enablement-in-chronic-heart-failure-reach-hf-in-wirral

Our patient coauthor KP has been a member of the REACH-HF study group for several years, having had a heart attack in 2008. KP reviewed and provided input to this manuscript and talks about his experience of receiving CR in a podcast from our 2015 *BMJ* clinical review, which is available at: https://soundcloud.com/bmjpodcasts/cardiac-rehab-patient.

Our patient advisers acknowledged the usefulness of the digital approach to cardiac rehabilitation, especially for those who struggle to get to centre based sessions. But they also highlighted the need for a more hybrid approach so that rehabilitation is as accessible as possible. The final manuscript was modified to reflect the patients’ views.

**A carer’s story, by Cindy Edgeler**

My husband had a heart attack in 2012, which also left him with heart failure. After having stents fitted on the day he was admitted to hospital, he was well looked after on the critical care ward, and then came home.

Although he received good aftercare, I felt that I had no support and was left to my own devices. I had many questions, worries, and fears. I would lie next to him at night while he slept and wonder if each breath would be his last. He would say: “Stop asking me if I'm alright.”

There was no one I felt I could talk to, and I needed reassurance.

A few months later, we were offered the Rehabilitation Enablement in CHronic Heart Failure (REACH-HF) home based cardiac rehabilitation programme and were presented with the Heart Failure Manual and Family & Friends Resource for Carers, which importantly linked me to a nurse facilitator. From the first meeting there was a stark contrast with our previous experience, in that it was not just about the care of my husband but also about my own wellbeing. The facilitator answered all of my questions, and I knew I had a point of call should anything give me cause for concern.

I recall that I was concerned about my husband’s breathlessness on one home visit, and the nurse facilitator explained by giving me an analogy of: “You wouldn’t expect your car to pull away in fourth gear, you start in first and move slowly through the gears.” This made complete sense to me then.

My husband has other comorbidities that affect his walking and general fitness, and this is why the home based exercises are so much better for our situation. We do the chair based exercise DVD together; we are able to pace ourselves and decide which level we start at. We continue with weekly weigh-ins to ensure we keep within healthy limits, and we found the progress tracker helped to monitor progress.

The patient manual itself was a tool that we used—and still use—together. There is a separate manual/resource just for caregivers, which I find very useful.

A video featuring Cindy and Chris Edgeler can be viewed at http://thebmjawards.bmj.com/showcase/.

**Additional educational resources for patients and carers**

American Heart Association: what is cardiac rehabilitation? www.heart.org/en/health-topics/cardiac-rehab/what-is-cardiac-rehabilitation
Additional information on home based cardiac rehabilitation. www.heart.org/en/news/2019/05/13/experts-urge-expansion-of-home-based-cardiac-rehabilitation
A telehealth cardiac rehabilitation programme for patients using a free app to use on a mobile phone/device. www.henryford.com/services/cardiology/support/cardiac-rehab/home-based-cardiac-rehabilitation
British Heart Foundation: online exercise videos. www.bhf.org.uk/informationsupport/heart-matters-magazine/activity/10-minute-workout
Online patient education sessions available in six languages. www.healtheuniversity.ca/en/CardiacCollege
A choice of a book or digital format of a home based programme for patients recovering from a heart attack or revascularisation. https://services.nhslothian.scot/TheHeartManual/Pages/default.aspx
A webinar on virtual rehabilitation and self-management techniques during covid-19 for people living with heart conditions. www.heartandstroke.ca/what-we-do/webinars/cardiac-rehab-during-covid-19

All resources are open access with no registration, except the digital format of the *Heart Manual*, which requires registration

**How this article was created**

We focused on new evidence on home based and telehealth based cardiac rehabilitation that would be of interest to patients, clinicians, and commissioners based on recent publications on the impact of covid-19 and our 2015 clinical review that was co-authored by HD, RST, and PD. RST is a contributor and editor for the Cochrane Heart Group and has led and conducted several systematic reviews of cardiac rehabilitation. These Cochrane reviews have been cited in various national guidelines, which we consulted when writing this article. We referred to annual reports of the National Audit of Cardiac Rehabilitation, which is led by PD, and the BHF website for statistics on coronary heart disease in the UK. We also consulted recent national statements and standards from the UK, US, and Europe, and HD used his personal archive of references.

## Notes

Acknowledgments: We thank Jemma Lough for help with technical editing of the manuscript, Cindy and Chris Edgeler for patient and care giver involvement, and the Wirral Community Cardiology Service for providing feedback from patients and practitioners involved in the REACH-HF home based programme.

Contributors: HMD conceived the article based on a clinical review he co-authored for *The* *BMJ* in 2015. He contributed to the literature review, drafting, and revising the article and approval of the final version. RST provided details from the various Cochrane systematic reviews that he has led and conducted. PD provided contemporary data from the BHF National Audit of Cardiac Rehabilitation. PD, RST, and SM also contributed to the literature review and drafting, design, and revision of the article. All authors, including patient and public adviser KP, reviewed and approved the final manuscript.

Provenance and peer review: Commissioned, based on an idea by the authors; externally peer reviewed.
